# Acquired Clotting Factor Deficits During Treatment with Asparaginase in an Institutional Cohort

**DOI:** 10.2147/JBM.S428159

**Published:** 2023-11-07

**Authors:** Vasiliki Papadopoulou, Giulia Schiavini

**Affiliations:** 1 Service and Laboratory of Hematology, Department of Oncology, Lausanne University Hospital, Lausanne, Switzerland

**Keywords:** asparaginase, clotting assays, acute lymphoblastic leukemia, NK-cell lymphoma, bleeding, thrombosis

## Abstract

We invariably see prolongation of activated partial thromboplastin time in patients treated with asparaginase in our clinical practice, but have noted that, contrary to hypofibrinogenemia and low antithrombin, clotting times’ prolongation by asparaginase is largely unreported in the literature and guidelines and is not widely known to clinicians. We report on aPTT prolongations in a small cohort of patients, and on their origin, as investigated by measurements of clotting factors, fibrinogen, and D-dimers before and after asparaginase administration. We observed significant reductions in FIX and FXI (median post-treatment values of 27 IU/dl and 52 IU/dl, respectively), confirming one previous observation. A decrease in FXII was less pronounced but contributed to the prolonged aPTTs (FXII has no effect on in vivo haemostasis). The factor deficits are not due to consumption, as evidenced by unchanged D-dimer levels, and are, therefore, probably caused by disturbed factor synthesis. Our observations and insights contribute to elucidation of the profile of clotting assays during asparaginase treatment, and thus, to optimally monitor for undesirable events or steer situations of therapeutic anticoagulation without the risk of suboptimal or excessive anticoagulation.

## Introduction

Asparaginase, in various formulations (L-asparaginase, Pegaspargase, Erwinase), is a backbone drug for the treatment of acute lymphoblastic leukaemia (ALL) and is also used in regimens for aggressive NK-cell leukemia/lymphoma. It has specific toxicities, including hypofibrinogenemia, low antithrombin levels, and a thrombotic tendency, with a possibly high proportion of CNS thrombosis.^1^ Hypofibrinogenemia and low antithrombin levels trigger administration of plasma/antithrombin products for severe deficits, according to different institutional practices, without solid evidence for cut-offs under which these products should be used.^2^

In our clinical practice, we invariably observe substantial prolongation of activated partial thromboplastin time (aPTT) in our patients with asparaginase, even in cases with low-normal fibrinogen levels, whose values alone do not justify aPTT prolongation. Moreover, in cases of concomitant heparin therapy, we usually observe excessive aPTT prolongations, with discordant aPTT and anti-Xa values. Prothrombin time (PT) prolongations are also present but are less pronounced. Contrary to hypofibrinogenemia and low antithrombin levels, prolongation of aPTT, or clotting times in general, by asparaginase, is not referenced in guidelines or clinical protocols, and we find it to be less common knowledge among clinicians. We were therefore interested to highlight this finding and to define the origin of aPTT prolongation and whether it mirrors a higher bleeding risk. To summarize the current knowledge on this topic, prolongation of coagulation screens has been published in the past, but only in very few studies dating four decades ago.^3–5^ Moreover, regarding individual clotting factor deficits under asparaginase treatment, to the best of our knowledge, the only references are ancient and come from a very limited number of patients. They have shown deficits of FIX and FXI during treatment (median measured values 62 and 48 IU/dL respectively) in a report on sixteen patients; in the same report, factors V, VII, VIII, X, II and XII were measured normal in most patients, with median values of 101, 150, 188, 116, 86 and 93 IU/dl, respectively, post-asparaginase. Factors II, IX, and X were measured low at 55, 47, and 63 IU/dL, respectively, in a second report we could find.^6^,^7^

## Methods

To characterize the aPTT prolongations we observe under asparaginase, we measured, in our clinical practice, clotting factors and D-Dimers, before and during the “asparaginase-phase”, in some of our ALL and NK-cell lymphoma patients treated with asparaginase-containing protocols in our institution from 01.09.20 to 01.09.22 (11 patients were treated in total, including 9 ALL and 2 NK-cell lymphoma; intrinsic pathway factors were measured in 6/11 cases, while factors II, V, VII, X were measured in 2/11 cases).

## Results

Ten out of 11 (10/11) patients showed aPTT prolongation; the results of screening and factor assays on the designated day after the start of the drug are shown in Table 1A and B. Highest aPTT values during the cycle are also shown. Patient 10, the only patient who received intramuscular pegaspargase, did not show prolonged aPTT or low fibrinogen levels during treatment. Three patients were anticoagulated, but their absolute aPTT value and aPTT ratio compared to the pre-asparaginase sample were excessive for their plasma heparin concentration measured by anti-Xa. Prothrombin times were moderately prolonged (Table 1B). We observed significant reductions in factors IX and XI with asparaginase in 6/6 patients in which these factors were measured, with a median decrease of 71% (median post-treatment value 27 IU/dL) for FIX and median decrease of 55% for FXI (median post-treatment value 52 IU/dL) (Table 1A). There were decreases of FVIII, but not to subnormal levels, as well as of FXII, with a median FXII reduction of 25% in the five non-inflammatory patients measured (all intrinsic pathway factors were measured in six patients in total, but one of the six patients was in inflammatory state at the post-treatment measurement, a state in which FXII levels decrease due to contact pathway activation, while FVIII levels increase as acute-phase reactant). The “common pathway” factors II, V and X, and the extrinsic pathway factor VII, were measured only in 2/11 patients, with average decreases in factors II, V, and X post-asparaginase of 29%, 40% and 50% respectively; no decrease in FVII was seen in these two patients. D-dimers, measured in all eleven cases, were not elevated compared to the pre-asparaginase status. Bleeding episodes were not observed.

**Table 1:** Coagulation Parameters Before Start and After Administration of Asparaginase in the Patient Cohort

**(A)**																									
**Patients/Diagnosis**		**aPTT (sec)**					**FVIII (IU/dl)**				**FIX (IU/dl)**				**FXI (IU/dl)**				**FXII (IU/dl)**				**Fibrinogen (g/l)**		
		**Pre**	**Post**			**AntiXa IU/mL**	**Pre**	**Post**		**D(FVIII)**	**Pre**	**Post**		**D(FIX)**	**Pre**	Post		**D(FXI)**	**Pre**	**Post**		**D(FXII)**	**Pre**		**Post**
**Pt 1**	T-ALL (26, m)	29	49 (d7) Max. 54 (d8)			0	176	113		−36%	90	26		−71%	105	56		−47%	85	66		−22%	1.8		1.3
**Pt 2**❖	B-ALL (20, f)	36	101 (d5) (Max.)			0.4	130	130		0	70	19		−73%	120	49		−59%	125	65		−48%	2.4		1.6
**Pt 3**❖	T-ALL (38, f)	29	67 (d3) Max. 70 (d6, antiXa 0.5)			0.5	229	168		−27%	120	57		−53%	130	75		−42%	140	105		−25%	2.1		1.4
**Pt 4**	T-ALL (55, m)	30	42 (d6) (Max.)			0	91	75		−18%	95	27		−72%	110	55		−50%	85	69		−19%	3.8		1.6
**Pt 5**	B-ALL (19, m)	27	51 (d10) (Max.)			0	175	125		−29%	80	23		−71%	95	25		−74%	75	53		−29%	2.8		1.0
**Pt 6**❖*	B-ALL (20, f)	24	89 (d10) (Max.)			0.15	119	155		+30%	115	36		−69%	105	37		−65%	175	37		−79%	1.5		4.1
**Pt 7**	B-ALL (64, f)	29	49 (d8) (Max.)			0	ND	ND			ND	ND			ND	ND			ND	ND			5.2		2.4
**Pt 8**	T-ALL (36, m)	31	77 (d22) (Max.)			0	ND	ND			ND	ND			ND	ND			ND	ND			1.8		1.5
**Pt 9**	NK (63, f)	35	127 (d12) (Max.)			0	ND	ND			ND	ND			ND	ND			ND	ND			5.5		1.4
**Pt 10**	NK (62, m)	27	37 (d21) (Max.)			0	ND	ND			ND	ND			ND	ND			ND	ND			2.1		1.8
**Pt 11**	T-ALL (36, m)	28	48 (d10) (Max.)			0	ND	ND			ND	ND			ND	ND			ND	ND			1.3		1.5
**(B)**																									
	**INR (PT sec)**		**Antithrombin (%)**			**FII (IU/dl)**				**FV (IU/dl)**				**FVII (IU/dl)**				**FX (IU/dl)**					**D-Dimers (ng/mL)**		
	**Pre**	**Post**	**Pre**	**Post**	**D(AT)**	**Pre**	**Post**		**D (FII)**	**Pre**	**Post**		**D(FV)**	**Pre**	**Post**		**D(FVII)**	**Pre**	**Post**		**D(FX)**		**Pre**	**Post**	
**Pt 1**	1.1 (11)	1.2 (d7) (12.5)	91	53	−42%	90	69		−23%	130	95		−27%	75	95		+27%	80	50		−38%		1440	1494	
**Pt 2**❖	1.1 (11.6)	1.2 (d5) (13.1)	62	33	−47%	ND	ND			ND	ND			ND	ND			ND	ND				394	288	
**Pt 3**❖	1.0 (10.5)	1.0 (d3) (11.4)	86	57	−34%	ND	ND			ND	ND			ND	ND			ND	ND				1023	1023	
**Pt 4**	1.1 (11.9)	1.2 (d6) (13.4)	92	62	−32%	ND	ND			ND	ND			ND	ND			ND	ND				1651	938	
**Pt 5**	1.1 (12.1)	1.5 (d10) (15.5)	81	53	−35%	63	41		−35%	100	47		−53%	48	51		+6%	80	31		−61%		1621	211	
**Pt 6**❖*	1.0 (10.8)	1.1 (d10) (12.2)	98	51	−48%	ND	ND			ND	ND			ND	ND			ND	ND				3128	1477	
**Pt 7**	1.0 (10.6)	1.1 (d8) (11.9)	82	56	−32%	ND	ND			ND	ND			ND	ND			ND	ND				3128	1477	
**Pt 8**	1.0 (11)	1.2 (d22) (12.4)	107	61	−43%	ND	ND			ND	ND			ND	ND			ND	ND				1941	2180	
**Pt 9**	1.0 (10.6)	1.3 (d12) (14)	102	41	−60%	ND	ND			ND	ND			ND	ND			ND	ND				1977	1249	
**Pt 10**	1.0 (11.0)	1.1 (d21) (12.1)	ND	53	ND	ND	ND			ND	ND			ND	ND			ND	ND				4376	1378	
**Pt 11**	1.1 (11.4)	1.1 (d10) (12.1)	88	41	−53%	ND	ND			ND	ND			ND	ND			ND	ND				277	312	

**Notes**: Pre-asparaginase samples: taken hours before first administration of the drug. Post-asparaginase samples: taken on designated day after start of the drug. Drug formulation was: L-asparaginase 6000 IU/m2 iv on days 1, 3, 5, 13, 15, 17, 19, and 21 for patients 1, 2, 3, 4, 8 and 11 (asparaginase effects persist for 72h and may be additive with repetitive doses) / L-asparaginase 6000 IU/m2 iv on days 1, 3, 5, 7, 9, 11, and 13 for patient 9 / Pegaspargase iv 2000 IU/m2 on d1 for patients 5 and 6 (half-life varying from 6 days −2 weeks) / Pegaspargase iv 500 IU/m2 on d1 for patient 7 (low dose, due to age) / Pegaspargase 2500 IU/m2 im on d1 and d13 for patient 10. Day 1 is 1st day of asparaginase administration for all patients, not the 1st day of the multi-agent regimen. The regimen for ALL patients (induction or late intensification) contained daunorubicin, vincristine, prednisone, cyclophosphamide, and asparaginase, according to GRAALL-2005.^16^ For NK-cell lymphoma cases: SMILE regimen for patient 9^17^ and pegaspargase-gemcitabine-oxaliplatin for patient 10. All patients had normal liver function except for grade I transaminitis in patient 9. **❖** Denotes patients therapeutically anticoagulated by unfractionated heparin at the timepoint of the post-treatment sample (1 PE and 2 DVT, all occurring before start of asparaginase). Factor measurement results were independent of the presence of heparin. All pre-treatment samples, as well as the post-treatment samples of all other patients, were drawn under prophylactic perfusion of 100 iu/kg heparin for/24h, with undetectable anti-Xa. * Patient 6 was in systemic inflammatory state (febrile bacteremia, without disseminated intravascular coagulation) at collection of the post-treatment sample. **A**. aPTT prolongation was observed in all patients except patient 10, with excessive prolongation in anticoagulated patients. Anti-Xa is shown for therapeutically anticoagulated patients post-asparaginase. Significant decreases in FIX and FXI are observed, with median reductions of 71% for FXI and 55% for FXI. FVIII is upregulated and FXII is downregulated in inflammatory states; therefore, they are not reliable to assess the effect of asparaginase in patient 6. These two factors did not show deep decreases post-asparaginase in the five non-inflammatory patients, with median decreases of 27% and 25%, respectively. Fibrinogen levels were invariably lower after treatment, except for patient 6, for whom it was elevated as an acute-phase reactant, and for patient 10, who showed no prolongation of coagulation screens. Highest aPTT values during the cycle are also shown (as Max.). **B**. PTs were moderately prolonged. There were no significant changes in D-dimers’ levels. Important decreases in antithrombin observed in all patients. Decreases in FII, FV, and FX, but not in FVII, were observed in two patients.

## Discussion

Based on our observations, we consider the acquired substantial deficits in FIX and FXI to be the main origin of the observed aPTT prolongations; these findings are in line with the two ancient reports cited in the introduction.^6^,^7^ Certainly, an isolated FIX at approximately 30 IU/dL, or, much less, an isolated FXI at approximately 50 IU/dL, would not be sufficient to provoke the aPTT prolongations observed, but the combined effect of multiple factor deficits, as well as of moderately low (low normal) fibrinogen levels, may produce visible aPTT prolongations. Moderately decreased levels of FXII also contribute to aPTT prolongation, given the sensitivity of the assay to this factor, which, however, does not play a role in in vivo hemostasis. Different asparaginase doses/schedules or formulations, as well as variable individual patient susceptibility, produce different coagulation factor nadirs, and the lowest levels are expected after consecutive doses. We observed no abnormal coagulation screens in the only patient who received pegaspargase intramuscularly, but we cannot conclude through only one case whether intramuscular administration attenuates the effects of the drug on coagulation. As for the moderate prolongation of prothrombin times, we suggest that they are brought about by moderate decreases in factors II, V, and X along with the moderately low fibrinogen, considering that we measured these factors in only two patients.

The mechanism of clotting factor depletion by asparaginase is unclear, as are the mechanisms of fibrinogen and antithrombin reduction, but research has shown that hypofibrinogenemia is not caused by peripheral consumption,^8^ and therefore a deficit of synthesis has to be assumed, and the same could be applied to the synthesis of multiple clotting factors. The non-elevation of D-dimer levels (**Table 1B**) after treatment in our patients excluded factor consumption, supporting this hypothesis. Indeed, asparaginase is known to alter hepatic metabolism, with the development of important hypertriglyceridemia, liver steatosis, and possible inhibition of protein synthesis, with unclear mechanisms probably related to depletion of glutamine and asparagine.^9^ The mechanism by which FIX seems to be more affected than other factors in our small cohort is unclear; however, the degree of acquired deficits should be precisely quantified with prospective data in larger samples, with measurements at defined time points and constant drug doses. FVIII, which seems less affected than other factors in our cohort, is synthesized mostly by endothelial cells; therefore, it is unknown whether the effect of asparaginase applies to its synthesis. As for FVII, which seems unaffected, we cannot generalize the findings as we measured it only in two cases.

Data on the bleeding risk with asparaginase in adults are limited, whereas a thrombotic risk related to asparaginase is rather widely reported;^10^ therefore, it is uncertain whether the observed factor deficits could provoke a bleeding tendency during treatment. In the large prospective MRC UKALL XII trial, an extremely low number of treatment-related deaths were reported in general, while the GRAALL 2005 prospective trial showed an incidence of grade 3–4 bleeding at 4% during induction.^11^,^12^ In large prospective trials in children, bleeding-related deaths were rare.^13^ A recent retrospective study on pegaspargase toxicities in 57 adults, published in abstract form, showed a frequency of all-grade bleeding episodes during induction at 7% and during post-induction at 10%, without available correlative data for fibrinogen levels or clotting times during induction.^14^ In another retrospective study, the incidence of any-grade bleeding was 5.3%, with no CNS bleeding, and was higher during induction, possibly due to concomitant thrombocytopenia.^15^ It is important to remember that not only clotting factors but also natural anticoagulants (protein C, protein S, antithrombin) are decreased by asparaginase,^1^,^7^ which can reduce the risk of bleeding. Thrombin generation potential was not decreased in asparaginase-treated patients in a relevant study, and interestingly, it was not substantially influenced by fibrinogen or antithrombin levels.^16^

## Conclusions

In summary, we report aPTT prolongations with asparaginase, secondary to acquired deficits of multiple clotting factors, due to globally impaired clotting factor synthesis, with factors IX and XI seemingly affected to a greater extent than others. A limitation of our results, except for the small number of cases in our series, is that factor measurements were not performed at highest aPTT/PT value of the cycle for all cases, so nadir factor values could be lower than those measured in some cases. Our observations confirm long-dating underemphasized data^5–7^ and are relevant for the understanding of the laboratory profile of patients treated with asparaginase, and therefore for their optimal assessment. Moreover, they suggest that, if aPTT is used to monitor therapeutic heparin in patients during the asparaginase phase of treatment, the aPTT ratio should be interpreted carefully; a denominator value taken pre-asparaginase can provide an excessive ratio and give impression of excessive anticoagulation. However, one could indeed ask whether a bleeding risk is present when very high absolute aPTT values (a result of the deficits of clotting factors and fibrinogen, in combination with anticoagulation) are found in this context. The levels of factors measured in our patients are not a contraindication to full-dose anticoagulation when the latter is indicated, and general experience with bleeding risk with asparaginase is benign; however, in cases of clotting assays implying severe factor depletion (which could result after consecutive asparaginase doses or in very susceptible patients), close clinical and laboratory monitoring for bleeding risk under anticoagulation is advisable, even more so in the simultaneous presence of thrombopenia due to myelosuppression.

## Data Sharing Statement

More detailed clinical/laboratory data are available upon request.
